# Accuracy of Acetabular Component Positioning Using Computer-assisted Navigation in Direct Anterior Total Hip Arthroplasty

**DOI:** 10.7759/cureus.4478

**Published:** 2019-04-16

**Authors:** Michael P Bradley, Jessica R. Benson, Jeffrey M Muir

**Affiliations:** 1 Orthopedics, South County Hospital, Wakefield, USA; 2 Clinical Research, Intellijoint Surgical, Waterloo, CAN

**Keywords:** total hip arthroplasty, direct anterior approach, computer-assisted navigation, cup position, accuracy

## Abstract

Background

Appropriate component positioning in total hip arthroplasty (THA) is imperative for long-term survivorship. C-arm fluoroscopy provides visual guidance in the direct anterior approach (DAA), but it is limited by qualitative properties. Conversely, imageless computer-assisted navigation systems (CAS) provide surgeons with intraoperative, three-dimensional (3D) quantitative measurements for cup position, although the accuracy of such systems has not been extensively addressed in the DAA. We evaluated the ability of an imageless CAS to deliver measurements for acetabular cup position with accuracy in the DAA.

Materials and methods

A retrospective analysis of 69 primary THA procedures was conducted. Acetabular cup position measurements (anteversion and inclination) obtained intraoperatively by imageless navigation were compared to standard, postoperative anteroposterior pelvic radiographic measurements. Statistical comparisons were made using the Bland-Altman technique.

Results

The mean difference between device and radiographic measurements for anteversion was 3.4° (standard deviation (SD): 4.1°; absolute mean difference (ABS): 4.2°), and 4.0° for inclination (SD: 3.6°; ABS: 4.3°). Bland-Altman analysis demonstrated excellent agreement; 93% (64/69) and 97% (67/69) of anteversion pairings fell within the statistical and clinical limits of agreement, whereas 94% (65/69) and 100% (69/69) of inclination pairings were within the statistical and clinical limits, respectively.

Conclusions

Measurements obtained intraoperatively for acetabular cup position using imageless navigation in the DAA are agreeable with the current clinical standard.

## Introduction

Component malpositioning increases the risk of accelerated component wear, joint instability, and impingement; it is also a leading cause of early dislocation and revision surgery following primary THA [1-2]. With an aging population and the associated increase in demand for primary and revision THA anticipated [3], optimizing the correct component positioning during THA is crucial.

Advocates of the direct anterior approach (DAA) report diminished dislocation rates that can be attributed to visualizing and confirming component positioning using intraoperative fluoroscopy, and to the preservation of anatomical stabilizers during surgery [4-5]. The DAA is currently gaining popularity for its association with improved postoperative outcomes such as decreased length of stay, patient-reported pain, postoperative resource utilization, risk of re-operation, and faster return of physical function and mobility [6-7]. Correct component positioning via the DAA is predominantly reported utilizing C-arm fluoroscopy [8], which can reduce variability in acetabular component positioning and improve implant placement with respect to the surgeon’s targeted position [8]. However, risks include musculoskeletal injury resulting from the prolonged use of heavy lead aprons [9], increased risk of infection as the C-arm is carted into the sterile field [10], and cumulative surgeon radiation exposure [11]. In turn, studies report that fluoroscopy is misleading [12-13]; correct positioning of the C-arm is crucial for delivering accurate representations of anteversion, and a 10° deviation in the C-arm tilt angle can lead to upwards of 9° error of perceived anteversion [12]. Pelvic tilt and extension prior to cup insertion also causes the C-arm to underestimate anteversion and inclination [12,14]. Such complications may arise from the two-dimensional (2D) nature of intraoperative fluoroscopy and likely contributes to the steep learning curve associated with such procedure [15]. C-arm fluoroscopy may, therefore, be limited by qualitative outputs, and be especially challenging for low-volume institutions and surgeons.

Computer-assisted navigation systems (CAS) conversely provide orthopaedic surgeons with a three-dimensional (3D) quantitative measurement for cup position, and numerous reports indicate an improvement in accuracy and precision of implant component placement, as well as a reduction in Lewinnek safe zone outliers [16], when compared to non-navigated THA [17-18]. Interestingly, the role of CAS in anterior THA remains less described. Early reports observed similar surgical time and improved surgeon precision with acetabular component positioning following the integration of CAS into the DAA surgical workflow [19]. In turn, imageless CAS may reduce current risks associated with fluoroscopy-guided THA as the systems perform independently of radiographic imaging [19]. However, further clinical evaluation of the accuracy and reliability of imageless CAS in the DAA is required. The present study evaluated the ability of imageless CAS to deliver accurate intraoperative measurements of cup position (anteversion and inclination) in the DAA as compared to postoperative anteroposterior (AP) radiographic measurements, the current clinical standard.

## Materials and methods

This study was a retrospective review of patients who underwent direct anterior primary THA, performed by a single surgeon (MPB), utilizing imageless, computer-assisted navigation (Intellijoint HIP, Intellijoint Surgical, Inc., Waterloo, ON, Canada). The study was reviewed and approved by a local institutional review board.

Patient eligibility

Patients eligible for inclusion in this study underwent primary THA performed with the DAA between October 2016 and August 2018 at a single community hospital. Specific inclusion criteria included the availability of postoperative anterior-posterior (AP) pelvic radiographs, as well as intraoperative utilization of the navigation device. Exclusion criteria for the present study included the intraoperative removal of the navigation device prior to obtaining final measurements that match the final implant geometry; instability in the native operative hip such that the attending surgeon was unable to reproduce and/or identify radiographic landmarks on postoperative radiographs; and radiographs unable to be properly scaled or measured in postoperative analysis.

Computer-assisted navigation

**Figure 1:**
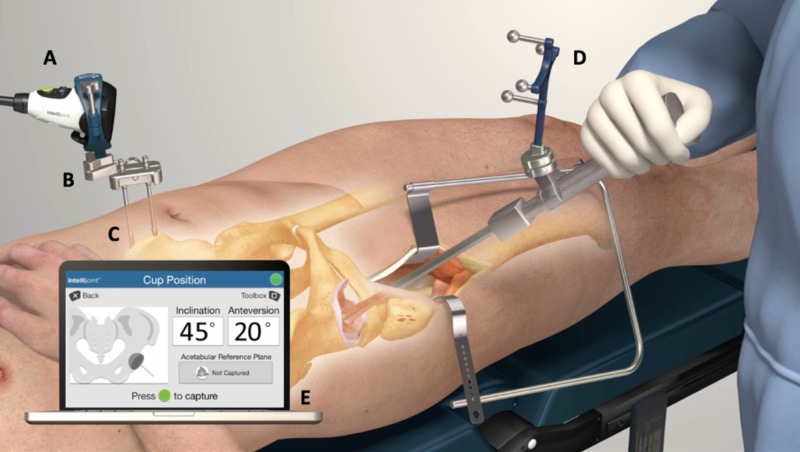
The Intellijoint HIP® mini-navigation system The camera (A) attaches magnetically to a pelvic platform (B) secured to the iliac crest of the patient’s pelvis via two pelvic screws (C). The device tracker (D) is magnetically fastened to the impactor. The camera captures positional changes of the tracker when registering the native orientation or while trialing the implant components. This positional information is then relayed to a workstation (E), located outside of the sterile field, for review by the surgeon.

The anterior application of the navigation tool has been previously described [20]. In brief, the system consists of a camera, probe, and tracker within the sterile field, and a workstation monitor located outside of the sterile field. When in use, the workstation computes the 3D positional relationship between the camera and tracker and provides this information in real-time on the display (Figure 1). Cup positional measurements are specifically acquired with the placement of the tracker onto the impactor. Final measurements are stored on the device and are accessible following surgery at any time.

Outcome variables

Acetabular cup positional measurements include anteversion and inclination. Intraoperative device measurements for acetabular anteversion and inclination were recorded by the navigation tool. Postoperative measurements for acetabular anteversion and inclination were obtained from standing, postoperative AP pelvic radiographs using TraumaCad software (Brainlab Inc., Westchester, IL) and the interischial line method. The mean difference and absolute mean difference (ABS) between the intraoperative device and postoperative radiographic cup positional measurements were recorded for each surgical case and defined as the device accuracy. A sub-group analysis was conducted on the study cohort to evaluate device accuracy stratified by gender.

Data collection and calculations

Device data regarding the final cup position was collected intraoperatively and stored by the navigation system. Patient records were reviewed to determine if any complications arose during surgery with the use of the navigation tool intraoperatively. Postoperative, standing AP pelvic radiographs were analyzed by a trained, independent observer and scaled using the known diameter of the femoral head implant. Radiographic measurements for cup position were reported as the mean of triplicate measurements.

Statistical analysis

Statistical comparisons were made with alpha set a priori at 0.05. The data were presented as the mean (standard deviation (SD)). Intra-observer validity for radiographic measurements was assessed using the intraclass correlation coefficient (ICC). Intraoperative device and postoperative radiographic cup positional measurements were compared using the Bland-Altman technique [21], a statistical analysis that determines the level of agreement between two measurement modalities. Specifically, the Bland-Altman technique generates an agreement interval that outlines the statistical limit where 95% of the difference of one method, as compared to the second method, falls. For the purpose of the present study, clinically relevant limits for anteversion and inclination were also included, set to +/- 10°, based on the Lewinnek safe zone [16]. Student’s t-test was used to evaluate device accuracy stratified by gender as well as demographic data, including age and body mass index (BMI), within the gender sub-groups.

## Results

Study cohort

In total, 69 cases were included in the analysis. The study cohort consisted of a mean patient age of 64.4 years (SD: 8.0; range: 52-89 years) and a mean BMI of 28.9 (SD: 4.7; range: 19 - 46.1). The right hip was the operative side in 63.7% (44/69) of cases. Males comprised 53.6% (37/69) of the sample population.

Cup position

**Figure 2:**
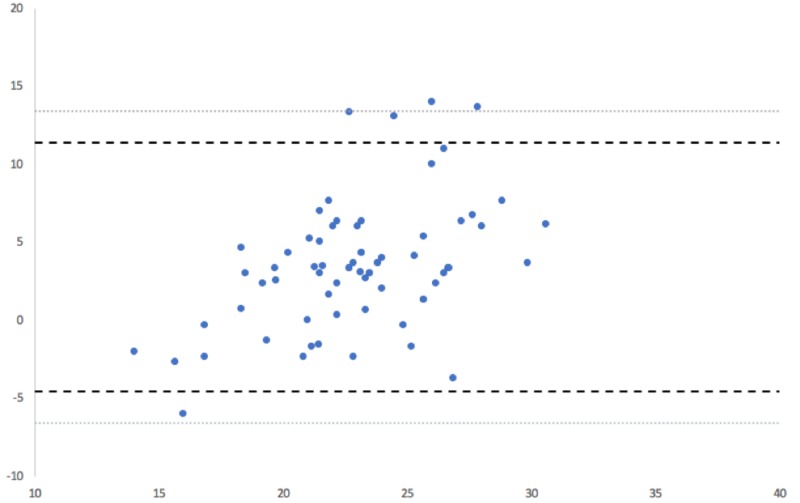
Bland-Altman analysis for anteversion Bland-Altman analysis showed excellent agreement for anteversion measurements obtained by the navigation tool and radiographically. 93% of measurements fell within the statistical limit for acceptance, and 97% of measurements fell within the clinical limit.

**Figure 3:**
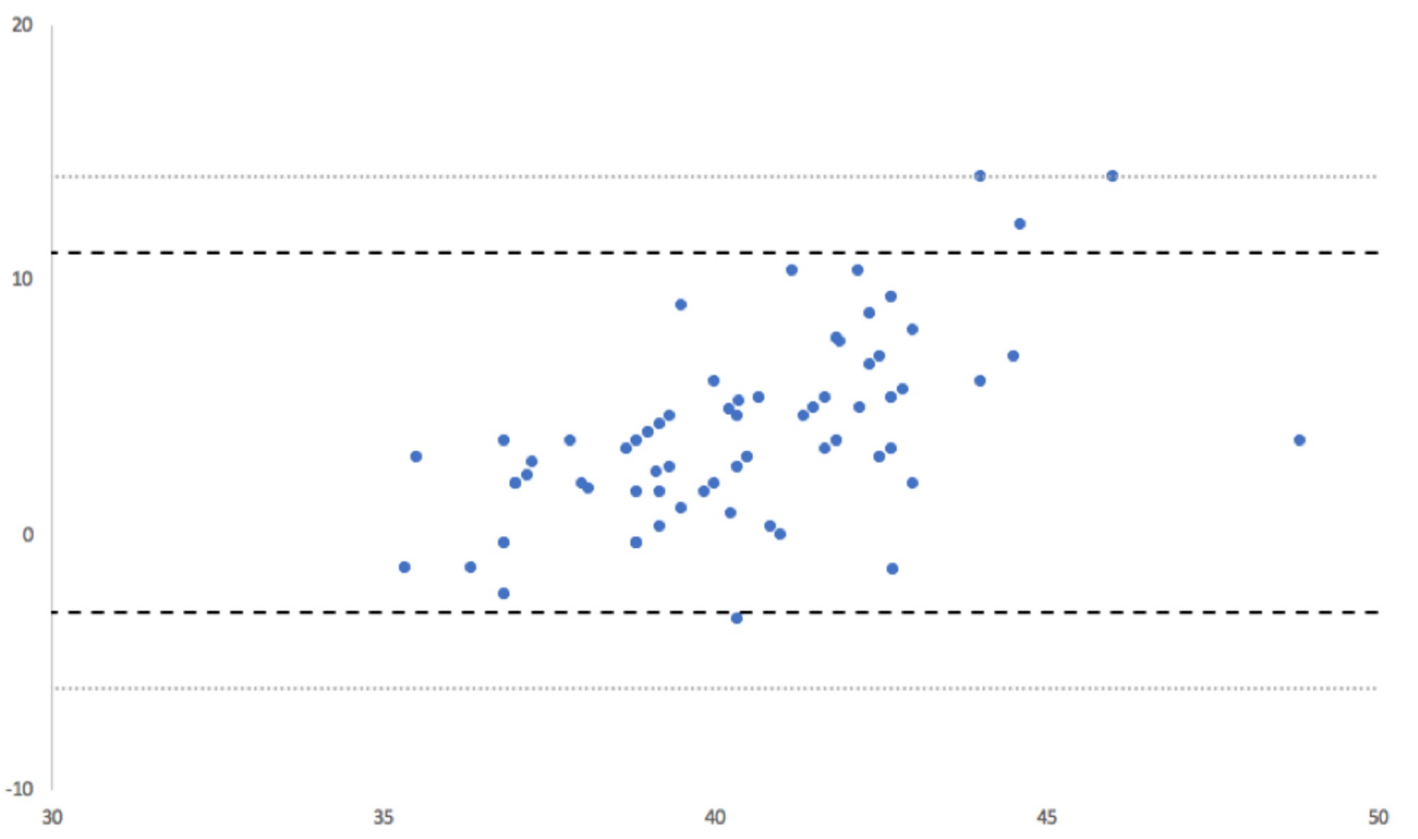
Bland-Altman analysis for inclination Bland-Altman analysis showed excellent agreement for inclination measurements obtained by the navigation tool and radiographically. 94% of measurements fell within the statistical limit for acceptance and 100% of pairings fell within the clinical limit for acceptance.

The ICC calculation for postoperative radiographic measurements demonstrated excellent intra-rater agreement (ICC = 0.99). Intraoperative device and postoperative radiographic cup positional measurements are summarized in Table 1. The mean anteversion angle was reported as 21.2° (SD: 3.0°) by the navigation tool and as 24.7° (SD: 4.7°) by postoperative radiographic measurements. In turn, the mean inclination angle as measured by radiographs was 42.5° (SD: 3.8°) versus 38.5° (SD: 2.1°), as measured by the navigation tool. Mean device accuracy for anteversion was 3.4° [SD: 4.1°; range: -6° to 14°; ABS: 4.2°] and 4.0° for inclination [SD: 3.6°; range: -3.3° to 14°; ABS: 4.3°]. A Bland-Altman analysis of intraoperative device and postoperative radiographic anteversion measurements demonstrated excellent agreement, as 93% (64/69) of anteversion pairings fell within the statistical limit of agreement, and 97% (67/69) of pairings fell within the clinical limit of agreement (Figure 2). A Bland-Altman analysis similarly demonstrated excellent agreement for inclination measurements, as 94% (65/69) of pairings were within the statistical limit and 100% (69/69) of pairings were within the clinical limit (Figure 3). Lastly, 85.5% (59/69) of radiographic acetabular cups were observed to be within 5° of the intraoperative device measurement of anteversion or inclination. In turn, 93% of radiographic anteversion and inclination measurements were within 10° of the intraoperative measurement.

**Table 1:** Summary of study cohort cup positional measurements SD: standard deviation

	Anteversion		Inclination	
	Intraoperative	Postoperative	Intraoperative	Postoperative
Mean (°)	21.2	24.7	38.5	42.5
SD (°)	3.0	4.7	2.1	3.8
Range (°)	15 – 28.7	13 – 34.7	34 – 47	34.7 – 53
Count	69	69	69	69

Subgroup analysis

A sub-group analysis stratified by gender was conducted on the sample cohort; demographic data and cup positional measurements for the same are summarized in Table 2. A statistical difference was not observed for age (P = 0.66) or BMI (P = 0.85) between male and female sub-groups. For the female sub-group, a mean device accuracy of 4.72° (SD: 4.1°; range: -2° to 14°; ABS: 5.0°) and 5.4° (SD: 3.9°; range: -1.3° to 14°; ABS: 5.6°) was calculated for anteversion and inclination measurements, respectively. In contrast, the male sub-group demonstrated improved accuracy, reporting a mean anteversion device accuracy of 2.3° (SD: 3.7°; range: -6° to 13.3°; ABS: 3.6°) that trended towards significance (P = 0.06), as well as a significant mean inclination device accuracy of 2.8° (SD: 2.8°; range: -3.3° to 10.3°; ABS: 3.3°) when compared to the female sub-group (P = 0.002).

**Table 2:** Demographic summary and cup positional measurements for gender sub-group analysis SD: standard deviation; BMI: body mass index

	Females			Males		
Count	32			37		
Demographics	Mean	SD	Range	Mean	SD	Range
Age (years)	63.8	8.9	52 – 86	65	7.2	53 – 89
BMI	29.0	5.7	19 – 46.1	28.8	3.6	20.7 – 37
Anteversion measurement	Mean	SD	Range	Mean	SD	Range
Intraoperative (°)	21.7	2.8	15 – 28	20.8	3.2	16 – 28.7
Postoperative (°)	26.4	4.7	13 – 34.7	23.2	4.1	13 – 33.7
Inclination measurement	Mean	SD	Range	Mean	SD	Range
Intraoperative (°)	38.2	1.6	34 – 41	38.7	2.4	35 – 47
Postoperative (°)	43.6	4.1	34.7 – 53	41.5	3.4	35.7 – 50.7

Follow-up outcomes

At 90 days post-operation, there were no dislocations or revision procedures reported within the study cohort. 

## Discussion

Correct component positioning is an important factor in ensuring long-term stability and survivability of the hip joint [2]. However, standard surgical protocols generally lack the tools to provide accurate, quantitative measurements for cup position intraoperatively. Although C-arm fluoroscopy is amenable to the DAA, this intraoperative tool is limited by its qualitative nature and requires precise positioning for reliability. The incipient adoption of navigation technologies in orthopedic practice for THA has demonstrated the improved accuracy and precision with which components are placed; however, these results are considerably less studied in the DAA specifically [17-18]. The present study evaluated the ability of an imageless CAS to provide accurate cup position measurements intraoperatively during THA facilitated by the DAA.

Component malpositioning during anterior THA is influenced by a number of variables, including but not limited to patient-specific anatomy, BMI, the incision size, and visibility [22-24]. While C-arm fluoroscopy provides visual cues to surgeons regarding component placement, the process is not without its limitations. A steep learning curve consisting of upwards of 100 cases is associated with the refinement of the intraoperative C-arm procedure [15]. A recent study by James et al [13] addressed misleading tendencies of C-arm fluoroscopy resulting from patient pelvic tilt. The study identified that 95.1% (39/41) of acetabular components were placed with unrecognized excess anteversion and inclination resulting from pelvic extension at the time of impaction, undetected by the C-arm. This is of concern, as Shah et al [14] studied intraoperative pelvic movement in the DAA and reported a pelvic tendency to move from a starting neutral position to a position of extension by the time of cup impaction. This was observed in 86.4% (19/22) of the study cohort. In turn, Jang et al., [12] demonstrated the importance of C-arm tilt angles for perceived and true cup position, with just 10° of caudal or cephalad C-arm tilt leading to a 9° and 10° error in perceived anteversion and inclination, respectively. Accurate fluoroscopy-guided THA is dependent upon the proper alignment of the surgical table, the patient, and the C-arm machine. Unaccounted for tilt or rotation across any of these factors can cause a positional error that may impede correct component positioning.

The present report addressed the ability of an imageless CAS to accurately measure cup position intraoperatively. In contrast to the standard C-arm workflow, where the correct positioning of multiple factors is assumed and in which cup position is only considered qualitatively, the intraoperative device used in the present report delivered quantitative cup positional measurements relative to the 3D anatomical reference planes of the patient, independent of alignment following registration and of imaging altogether. Cup position was reported with intraoperative patient movement taken into consideration, a potentially important feature as previous studies have shown that the pelvis rolls by as much as 9°. prior to cup impaction in the DAA [14] and 1° of pelvic roll can lead to 0.64° and -0.20° changes in anteversion and inclination, respectively [25]. Indeed, the present report demonstrated device accuracy to within 3.4° and 4.0° of postoperative radiographs for anteversion and inclination, respectively. The results of the present study are consistent with a recently completed evaluation of the accuracy of this same device in the DAA, which noted differences of 3.9° and 5.5° between navigation and postoperative radiographs for inclination and anteversion, respectively (personal communication; Alexiades 2018). Interestingly, our results are also congruent with those reported for robotic navigation in the DAA, as Redmond et al [26] noted robotic navigation cup position accuracy to within 3.5° and 3.9° of postoperative radiographs. The robotic navigation study also observed 92.7% and 97.6% of radiographic measurements within 10° of robotic navigation measurements for inclination and anteversion, respectively. Similarly, we observed 93% of radiographic cup measurements to be within 10° of the intraoperative CAS measurement. 

An observational sub-group analysis was conducted to consider device accuracy as stratified by gender. Interestingly, cup positional measurements in the male sub-group showed improved device accuracy when compared to the female sub-group. Although not examined in the current study, this may be in part due to soft-tissue distribution differences between males and females. Increased hip and thigh adipose distribution in females could account for an increased risk of anterior superior iliac spine (ASIS) registration error. Indeed, Richolt et al [27] reported a mean 2.8° anteversion error resulting from soft-tissue thickness overlying the ASIS. Despite no significant difference in BMI between male and female sub-groups (P = 0.85), the present report demonstrated a 2.42° difference in anteversion device accuracy between the male and female sub-groups. Further clinical analysis is required.

The limitations of the present study include the retrospective nature in which study data were analyzed; the observational data contained in this report was not powered and decreases the veracity with which conclusions can be derived. In turn, results were not matched with a control cohort; however, the main purpose of the present study was to simply address the accuracy capabilities of imageless navigation in the DAA. This was made possible with the use of postoperative AP pelvic radiographs for comparative analysis, the current clinical standard for cup position assessment. It is important to note that the device registers and displays cup positional data according to the patient’s supine coronal plane. As such, this study was further limited by an inability to assess postoperative cup position in the supine coronal plane, as standing AP pelvic radiographs were acquired for each patient. Several studies have highlighted significant changes in acetabular orientation resulting from different functional positions [28-29]; this may have contributed to the cup position accuracy error between the intraoperative and postoperative measurements observed in the present study.

## Conclusions

In the present study, an imageless navigation tool accurately delivered intraoperative measurements of acetabular anteversion and inclination in the DAA. The capabilities of imageless CAS may surpass the capabilities of traditional fluoroscopy-guided THA and provide the added benefit of reduced radiation exposure to the patient and surgeon; further clinical analysis is required.
